# A FAIR-Compliant Management Solution for Molecular Simulation Trajectories

**DOI:** 10.1021/acs.jcim.4c01301

**Published:** 2025-02-20

**Authors:** Andreas Vitalis, Steffen Winkler, Yang Zhang, Julian Widmer, Amedeo Caflisch

**Affiliations:** Department of Biochemistry, University of Zurich, Winterthurerstr. 190, 8057 Zurich, Switzerland

## Abstract

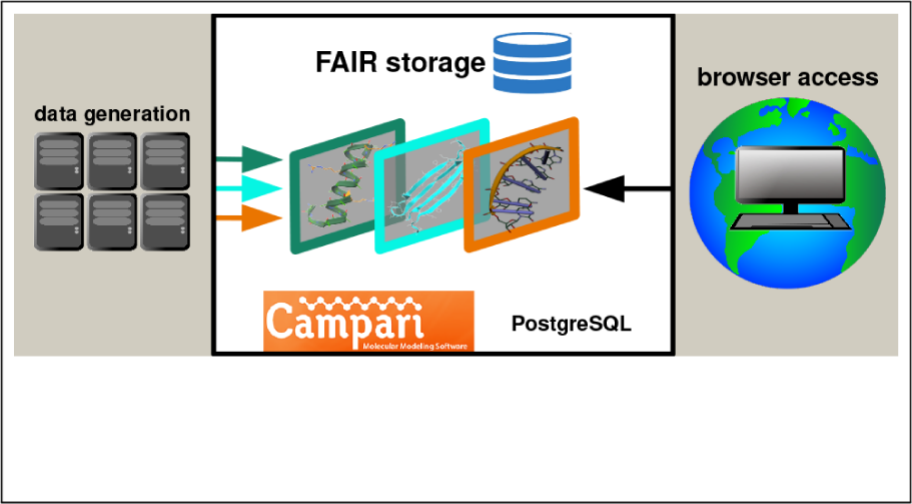

Simulation studies of molecules primarily produce data that represent the configuration of the system as a function of the progress variable, usually time. Because of the high-dimensional nature of these data, which grow very quickly, compromises are often necessary and achieved by storing only a subset of the system’s components, for example, stripping solvent, and by restricting the time resolution to a scale significantly coarser than the basic time step of the simulation. The resultant trajectories thus describe the essentially stochastic evolution of the molecules of interest. Maintaining their interpretability through metadata is of interest not only because they can aid researchers interested in specific systems but also for reproducibility studies and model refinement. Here, we introduce a standard for the storage of data created by molecular simulations that improves compliance with the FAIR (Findable, Accessible, Interoperable, and Reusable) principles. We describe a solution conceived in PostgreSQL, along with reference implementations, that provides stringent links between metadata and raw data, which is a major weakness of the established file formats used for storing these data. A possible structure for the logic of SQL queries is included along with salient performance testing. To close, we suggest that a PostgreSQL-based storage of simulation data, in particular when coupled to a visual user interface, can improve the FAIR compliance of molecular simulation data at all levels of visibility, and a prototype solution for accomplishing this is presented.

## Introduction

With the advent of globally and permanently accessible, long-term storage of data, the reusability of scientific data sets has become a focal point of interest in research communities. In 2016, the publication of the FAIR (Findable, Accessible, Interoperable, Reusable) principles^1^ offered a guiding framework for addressing these challenges, which has since been adopted, albeit often loosely, by journals and grant agencies around the world. In commercial practice, data are often viewed as capital and, consequently, are not generally shared. Conversely, the spirit of academic research encourages data sharing,^2^ and the more pressing issue is often the methodologies to effectively accomplish the sharing. Maintaining accessibility can pose a challenge for research groups. However, the emergence of open data repositories such as Zenodo, Figshare, GitHub, or Dryad, and data-centric journals such as Scientific Data or GigaScience has provided a boost in this regard and simultaneously made data sets easier to find.^3^ The biggest current challenge is probably in the combination of findability/accessibility and interoperability.^3^ Frequently, the metadata associated with a given primary data set are not sufficiently rich to allow working with these data without additional information, to be gathered, for example, from journal articles or private communications. The complications can be manifold, including the interpretation of the primary data, the environmental conditions, the understanding of outliers, the recognition of the limitations of the instruments used for measurements, etc. To give just a single example, in animal studies, it can be important to be able to diagnose observer bias introduced by different technicians performing the same assessment.^4^ To fully comprehend the data, this information must therefore be recorded and later indicated somewhere in the metadata.

Molecular simulations^5,6^ are mostly performed by integrating suitable equations of motion, using computer models of real systems at the molecular scale to understand states and processes at very high temporal and spatial resolution. There are three main reasons why interoperability and reusability, for which they must be both findable and accessible, are particularly important for simulation data. First, due to the scale of the systems under study, they behave stochastically, which means that inferences drawn from molecular simulations are susceptible to high uncertainty (most commonly known as the “sampling problem”).^7−12^ This results in inferences that carry ambiguity, which provides a clear motivation for robustness checks and cross-comparisons with data obtained on similar systems. Second, the computational models are incredibly detailed, too detailed in fact to analyze them in their totality.^13−16^ Consequently, published results inevitably discard and/or overlook processes that might be of interest to others and cannot be easily regenerated without a significant investment of both resources and expertise. Third, as a peculiar aspect of these data, they can be visualized dynamically (usually representing trajectories evolving in time) using suitable molecular viewers.^17−20^ While this type of data interaction entails many pitfalls, it is an indispensable tool in the arsenal of most practitioners in the field.

The vast majority of those simulation data sets that form the basis of a publication are not directly available at all. There are many reasons for that, some of which are largely historical by now. In our view, the biggest obstacle is that the data are surprisingly difficult to interact with and lack essential metadata.^21^ Partially, this is due to the format: simulation data sets are generally stored in binary files of three common varieties,^22−24^ none of which are interpretable at all on their own, and all of which require specialized software to interact with. In contrast, fully annotated and human-readable file formats like PDB or mmCIF are much too unwieldy to be practically useful.^25^

This composite of issues has inspired a number of initiatives to share simulation data on the web. These are comparable to global repositories for other data, such as the PDB for static structures or UniProt for protein sequence/function information. They face the universal challenge of obtaining long-term funding, and early initiatives^26,27^ probably fell victim to this requirement. They also face the 3-fold challenge that simulation data are poorly standardized, very large, and difficult to curate. These factors combined likely contribute to the choice for most of the more recent databases to limit their scope, e.g., to specific classes of systems, or to be restricted to simulations carried out with a standardized protocol. Examples include GPCRmd,^28^ ATLAS,^29^ or databases focused on COVID targets^30^ and nucleic acids.^31^ A completely general database faces challenges from all three factors: one of the most difficult factors is curation. At present, it is not clear to us how it will be feasible, in practice and on a resource budget, that a general, user-populated repository can remain interpretable or interoperable. For example, a clash detection algorithm is a largely unequivocal and automated validation tool for a static structure but not generally applicable to simulation data, since it fails to account for scenarios like free energy growth calculations, metadynamics, etc.

Because a globally visible server is almost always tied to specific modalities of the hosting platform, there is generally a codesign of software and the surrounding infrastructure (both software and hardware). This means that the implementation choices are not homogeneous. They also host, primarily, original files with the metadata layer provided by the server and populated during upload or curation. In contrast, this article describes a technical standard we propose and an implementation to homogenize and simplify the upholding of FAIR principles for molecular simulation data. The proposed standard addresses the metadata issue, at least in part, and couples it to a universal database management system via the Structured Query Language (SQL). This is useful for several reasons. First, it allows a stronger link between the writing of trajectory data and associated metadata, and no individual, user-visible files are created. Second, it allows linking related trajectories together, for example by system, but also in complex scenarios like parallel trajectory ensembles from, e.g., replica exchange.^32^ Third, as a standard support technology for web applications, SQL offers straightforward routes toward barrier-free access through the browser as needed and desired. Fourth, the reliance on SQL also implies that searchability, for example, keywords, system properties, simulation properties, etc., can be implemented at the data level. Such a standard can constitute the backbone of a public repository serving a global community, of a local repository in a company or university department, or even of a personal database and “lab book” for simulation data.

In this research, we first lay out important metadata that simulation data must include to become interpretable. We then describe the hierarchical design of an SQL database that also handles more general classes of simulation data (trajectory ensembles). This is followed by a description of a reference implementation, including suggested query logic, in the software packages CAMPARI^33^ and PostgreSQL/libpqxx^34^ and some limited performance testing. We finally conclude with a discussion on where and how the proposed standard can contribute to a FAIR-driven approach toward molecular simulation data.

## Methods and Results

### Requirements

Computer simulations of molecular systems generally require specifying the model to describe the evolution of the system (the “force field”), the system itself and its degrees of freedom, the sampler and the software that implements it (which define an approximate thermodynamic ensemble), and a boundary condition.^35^ Once simulation data have been generated, their extent (number of simulations, length) must be known, as well as the relationship between saved data and the full raw trajectories (generally, data are saved at reduced time resolution, preserving only a subset of coordinates that are deemed of interest). In the following, we restrict the discussion to classical simulations with fixed molecular topologies, but many of the concepts generalize straightforwardly.

#### System

The system will generally be a set of elementary particles (atoms) composed of a mix of (short) polymers, solvent, ions, etc. In order to understand exactly what these atoms are meant to represent, an annotated structure file serving as a template will ordinarily accompany binary trajectory files. This information must be part of the database, and it must be unequivocally linked with coordinate information. For every atom, the metadata should make it clear what role that atom plays in the system. This is essential for recapitulating the assignment of model parameters, which are impractical to store in the database (see the section Model).

In addition, the system must be contained in a finite volume; i.e., it requires a boundary condition. The most common choice by far is to use 3D periodic boundary conditions, which virtually replicate the system in all directions. This means that multiple images of molecules can matter and that conventions need to be obeyed even for simple issues like how to represent coordinates. For example, all atoms can be forced to reside in the same unit cell (which can effectively split molecules) or the integrity of molecules can be prioritized instead. It is not generally possible to convert between the two if the boundary condition is not known exactly, which is why unit cell dimensions are the only metadata explicitly contained in all binary trajectory file formats.

Some simulations produce systems of fluctuating composition (e.g., when simulating in the grand-canonical ensemble), and in those cases, they must be forced into a fixed composition logic. The normal way to do so is to define a superset system that includes ghost or buffer particles and to annotate the physical status of every particle (ghost or present) unambiguously. Any other approach is, in our experience, highly impractical.

#### Model

If the composition of the system is fixed, then the model generation, which in a classical force field consists of assigning interaction potentials and parameters to (tuples of) atoms, should be an exactly reproducible process. It is challenging to include these standard assignments in the database for the following reasons. First, parameters are assigned to (2–5)-tuples of atoms: this creates a new hierarchy that is not needed elsewhere. Second, the parameters make sense only for the entire system, but the stored simulation data will generally only contain parts of the system (stripping solvent molecules, for example). Third, the parameters and especially the interaction functions are not necessarily exposed to the user in the software running the simulations. This means it might be hard to find the relevant details even for experienced practitioners, as they will be contained only in essentially unrelated publications. For these reasons, in papers, the field uses shorthand abbreviations to refer to standard models, and we suggest using the same precise but succinct language in the metadata.

The main exception occurs when a part of the model has been modified or generated by hand. In classical simulations, this most often occurs in two scenarios: either when simulating molecules that are not described by the parent force field or when using custom restraint potentials. For the former, new parameters must be added, and these should be described exhaustively in the metadata, probably even when an exactly reproducible protocol, such as CGenFF,^36^ was followed. The latter is best explained in an example: suppose a simulation incorporates a set of upper distance restraints derived from NMR experiments.^37,38^ If the resultant simulation data are meant to be interpretable, then the exact specification of these custom restraints must be given, for which interoperability can be difficult to maintain.

#### Sampler

When the system and model are defined, they need to be simulated to obtain the type of data that this manuscript is concerned with. A software package will implement a numerical scheme, and this numerical scheme defines an approximate thermodynamic ensemble. The sampling scheme alters a well-defined set of degrees of freedom, which need not be those of the elementary particles (atoms). For example, someone interacting with the data who is interested in vibrational frequencies must know whether the corresponding bond lengths were constrained^39−41^ during the simulation or not. Similarly, any analysis of the data regarding free energies hinges on knowing which thermodynamic ensemble was targeted and whether it was modeled accurately in terms of fluctuations (e.g., inferences that are sensitive to ensemble fluctuations cannot be computed when the weak coupling scheme was in use as it creates an ill-defined ensemble).^42^

Advanced sampling strategies are a second concern: there are methods that (i) do not (just) alter the model but introduce bias through the choice of starting positions of many simulations (so-called “adaptive sampling”); or (ii) alter the model but by using a dynamically constructed bias potential (this includes “flat-histogram” methods like metadynamics).^43−46^ Neither type of simulation data is interpretable without sufficiently rich metadata. For metadynamics and related techniques, all of the required information might not even be exposed by the software, which is a caveat. Even relatively simple parallel techniques like replica exchange,^32^ where simulations periodically swap conformations, expose that classical trajectory files are not well-suited to create interoperable data sets. The swap history is not contained in the raw data, which means it can get lost independently, damaging or even destroying the value of the actual data set in the process.

The last concern is with the software itself. Software inevitably contains bugs that are resolved eventually. Similarly, implementations of algorithms can evolve over time, for example, due to performance enhancements. While such changes are well-documented in the online resources and/or the version management system, they are not common knowledge. Thus, the FAIR principles suggest that, at the very least, the exact version of the software used is contained in the metadata, along with particular caveats that the authors are aware of.

#### Omissions

Running molecular simulations normally implies a thermodynamic ensemble. For example, the results from a microcanonical simulation can be hard to interpret without having associated potential and kinetic energies. These and similar ensemble quantities are not normally recomputable for the following reasons: (i) velocities are needed; (ii) the stored trajectories are subset trajectories (for example, they omit solvent); and (iii) they require full knowledge of the interaction model in use, which includes both the force field and a truncation scheme. The truncation scheme, in particular, is extremely difficult to recapitulate consistently across different software packages. Unless this scheme is contained in the database, ensemble parameters such as potential energies would have to be deposited directly, yet they would remain challenging to interpret and be of limited use. To give an example, practitioners who are interested in splits of these values across subsystems, e.g., to compute the (direct) interaction energy of two polymers, will not be able to extract this information from the deposited numbers. Such requests are not possible to anticipate in general, which is a limitation. These deficiencies could only be overcome by allowing recomputations of these values, which, aside from the downsides of massively bloating the data footprint when storing full systems and velocities, would often be only approximate. Thus, we decided not to have dedicated fields for ensemble variables (like pressure, temperature, energies, etc.) in the database and allow their inclusion only at the level of text-based metadata. The same is true for analysis quantities, which are all parameter-dependent. For simple ones, SQL functions offer on-the-fly solutions.

### Implementation

Relying on SQL offers the advantage that we can design data relations and template queries that link pieces of information together: this is exactly what is missing when working with a set of binary trajectory files, annotated structure files, and other metadata (such as text from a journal article). Operations such as reordering snapshots in a trajectory, reordering atoms within snapshots, or splicing together snapshots from separate files are tedious to perform on dcd- and xtc-files, less so on NetCDF files, which at least allow straightforward near constant-time indexing in the snapshot dimension. CAMPARI offers conversions between all of these formats, additionally including pdb-files. It is also able to perform operations on trajectory ensembles such as changing a replica-exchange data set from containing swaps of conditions to containing swaps of conformations instead.^47^ It is therefore a natural choice to use CAMPARI and link it to the standard programmatic API of an open-source SQL database. For the latter, we chose PostgreSQL, which is accessible through a C++ API called libpqxx (https://pqxx.org/libpqxx/). The core functions performing database operations in CAMPARI are written in C++ and interfaced from Fortran using standard means. These core functions have also been isolated into their own reference library at https://gitlab.com/CaflischLab/TrajPSQLmod, which is documented (in-code) and offers Python bindings. This library lacks all automated metadata extraction, however, which is particularly relevant for atoms.

**Figure 1:**
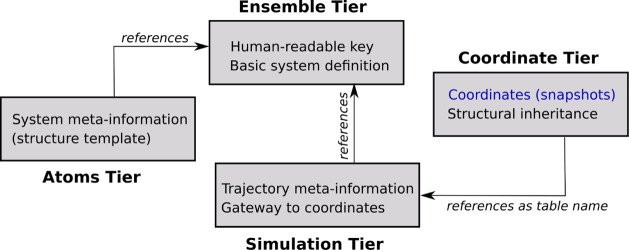
Layout of database. The different tiers are explained in detail in the text. Briefly, the atoms tier is needed to map atoms with specific roles to individual coordinates, the simulation tier is needed to understand the modalities under which coordinates were generated, and the ensemble tier links all tiers together. Traditional trajectory files in binary formats contain only the variable coordinate and box information (blue font). “Structural inheritance” refers to knowledge of the continuity of geometric evolution in a trajectory, which can be broken by, e.g., replica-exchange swaps.

We next describe the layout of the database, which is structured into four separate tiers, as shown in Figure 1.

At the top level, there is the ensemble tier, which is a container structure (mapping table), whose primary role is to link the information from the other tiers using a unique key. An ensemble is unified by referring to a system of fixed composition, which is expressed by a set of associated atoms that embed their own metadata. For this system, which is retrievable from the atom tier using the ensemble key, any number of groups of simulations might have been performed, and these are contained in the simulations tier. They can cross-reference each other, for example, to highlight roles in a parallel simulation ensemble such as replica exchange. Lastly, there is the coordinate tier, which is a set of tables, each of which contains data for exactly one simulation.

We now describe the tiers in detail.

#### Ensemble Tier

The ensemble tier, Table 1, is a container structure that provides a map between an interpretable name (“ens_key”, which must be unique and should not contain special characters except underscores and hyphens) and a numerical ID that forms the actual SQL key, “ens_id”. The latter is the primary lookup and linking mechanism to connect the tiers. The column “nratoms” is a redundant sanity check to detect database corruption (there must be exactly “nratoms” rows in the “atoms” table matching the given “ens_id”). The composition of the stored system is contained in “residues”, which mimics a CAMPARI sequence file, which in turn resembles a SEQRES entry in the Protein Data Bank (PDB) standard.^48^ This information is used not only as a convenience and cross-check mechanism (in principle, the atoms table contains this information) but also as a query target, for example, when trying to find particular sequence patterns.

**Table 1:** Ensemble Tier

Column name	PostgreSQL variable type	Array length	Key status
ens_id	INTEGER	N/A	Primary
ens_key	TEXT	N/A	None
nratoms	INTEGER	N/A	None
residues	TEXT	Dynamic	None
timestamp	TIMESTAMP	N/A	None

Finally, the timestamp is a specific PostgreSQL data type that allows sorting and querying by deposition time.

#### Simulation Tier

The simulation tier, Table 2, encompasses the meta-information for a given homogeneous subset of data associated with an ensemble. Through the column “ens_id”, it is linked to the ensemble tier and contains the information needed to find the tables that will hold the actual structural data for this subset. In the simplest scenario, each row in this single table corresponds to a single trajectory and provides the most critical details for how to interpret the data, such as the box shape and the sampling interval.

**Table 2:** Simulation Tier

Column name	PostgreSQL variable type	Key status
ens_id	INTEGER	Foreign
sim_id	INTEGER	Primary
sim_rank	INTEGER	None
sim_parent	INTEGER	None
sim_summary	TEXT	None
sim_software	TEXT	None
nratoms	INTEGER	None
equilibration_steps	INTEGER	None
snap_interval	INTEGER	None
box_shape	INTEGER	None
box_periodicity	INTEGER	None
ensemble_type	INTEGER	None
sampler_type	INTEGER	None
parallel_mode	INTEGER	None
united_atom_model	INTEGER	None
deposit_mode	INTEGER	None

When simulations are part of trajectory ensembles, such as in a replica-exchange run, one of the replicas serves as the parent (master), signified by its “sim_id”, which the associated replicas store in the field “sim_parent”. It will often be required to preserve the original order of the replicas, and this is what the “sim_rank” field is for, which is populated independently for each such ensemble of trajectories. The associated structural data appear in separate tables named systematically as “snapshots_(sim_id)_(sim_rank)”. Aside from the largely self-explanatory attributes (see below), additional meta-information is packaged as a free-text field, “sim_summary”. While we can imagine specifying a much larger number of columns to capture possible details of simulations, this quickly becomes impractical: first, all information not relevant during deposition (like details of the Hamiltonian) will be prone to errors that are very hard to curate; second, not everything can be anticipated (for example, custom restraint potentials on custom reaction coordinates used to improve sampling); third, many settings would require defining a standard first to avoid issues with differing units or text-based recognition (for example, “AMBER” vs. “Amber”). All further details should be contained clearly and as findable as possible in “sim_summary”. We anticipate that, most often, searching this text would be done to find simulations of a particular system, but in principle, the text should be structured so that any relevant property can be searched for using reasonable effort. Adding a full-fledged text search engine on top of this field will probably be necessary for making full use of it, but this is beyond the scope of the current work.

Naturally, constructing queries for the predefined attributes is much simpler and much more precise. They are as follows (some of them use an integer code inherited from CAMPARI key-files): The number of atoms, “nratoms”, of the associated system is self-explanatory. The “equilibration_steps” and the “snap_interval” report how many steps of the original simulation were discarded initially and what the saving frequency was. The “box_shape” is either 1 (rectangular cuboid), 2 (sphere), 3 (cylinder), or 4 (triclinic), while the “box_periodicity” ranges from 0 (no periodicity) to 7 (3D periodic) with one or two periodic dimensions in the order of *z*, *y*, *x*, *yz*, *xz*, and *xy* occupying values from 1 to 6. Spheres must be aperiodic, and cylinders must be aligned to one of the coordinate axes and have at most one periodic dimension, while partial periodicity for triclinic cells refers to the order of box vectors when speaking of *x*, *y*, and *z*. The “ensemble_type” is either 1 (canonical, NVT), 2 (microcanonical, NVE), 3 (isothermal–isobaric, NPT), 5 (semigrand), or 6 (grand canonical, μVT). For grand ensembles, where particle numbers can fluctuate or change identity, the database uses the strategy to store fixed particle numbers (including buffer particles) but indicates their physical presence at any given time by shifting their coordinates. Regarding the simulation approach, the database distinguishes various parallel schemes: “parallel_mode” is 0 for none, including brute-force multicopy sampling, 1 for replica exchange, 2 for progress index-guided sampling (PIGS), 3 for parallel metadynamics or Wang–Landau schemes, 4 for parallel simulated annealing, 5 for any other adaptive sampling scheme (unmodified Hamiltonian), and 6 for conformational reservoir-type or library-based sampling approaches. For samplers, we follow largely the CAMPARI keyword definition (“sampler_type” is 1 for Monte Carlo, 2 for Newtonian dynamics, 3 for stochastic dynamics, 4 for Brownian dynamics, and 5–7 for hybrid schemes). Lastly, “united_atom_model” specifies whether hydrogen atoms are present on polymer entities (0 for all, 1 if aliphatic ones are missing, 2 if only polar ones are retained, and 3 for other coarse-grained particles), and “deposit_mode” indicates whether (1) or not (2) a simulation was deposited as it was running. All of the above standards based on integers are easily extensible in the future. At present, the CAMPARI documentation is the authoritative source for the current version of the standard (in case extensions are implemented in the future).

#### Atoms Tier

While it has the most columns, Table 3 has the simplest structure. Its purpose is to be able to create annotated structure files (like PDB or Tripos mol2) from the database, which combine meta-information about the structure with the actual coordinates. Here, the meta-information not found in the coordinate tier are those that describe atomic properties (name, element, etc.) and the role of atoms in the polymer/system (residue names, molecule numbers, topological information, etc.). These properties are fundamental for a visualization engine to render them in the ways that have become standard in the field (such as rendering nitrogen atoms in blue, or showing covalent bonds as sticks). To construct an ATOM/HETATM line of a PDB file, one would use the fields “keyword”, “atom_seq”, “atom_name”, “resid_name”, “chain_seq”, “resid_seq”, *x*, *y*, and *z*-coordinates, “occupancy”, and three extra fields. Because the database deals with exactly defined structures, there is no need or room for insertion codes or alternate location identifiers. The coordinates are part of the coordinate tier while the occupancy field is redundant (always 1.0). The three extra fields are normally the (undefined) temperature factor, which can be filled with a parameter of choice (like “partial_charge”), an element symbol, which must be derived from mass or name and is not stored directly, and a net charge indicator, which can be derived from “formal_charge”.

**Table 3:** Atoms Tier

Column name	PostgreSQL variable type	Unit	Key status
ens_id	INTEGER	N/A	Foreign
atom_id	BIGINT	N/A	Primary
atom_seq	INTEGER	N/A	None
sys_seq	INTEGER	N/A	None
atom_name	CHAR(5)	N/A	None
resid_seq	INTEGER	N/A	None
resid_name	CHAR(4)	N/A	None
chain_seq	CHAR(2)	N/A	None
keyword	CHAR(6)	N/A	None
occupancy	DOUBLE PRECISION	Fraction	None
tempfactor	DOUBLE PRECISION	N/A	None
biotype	INTEGER	N/A	None
molecule_id	INTEGER	N/A	None
mass	DOUBLE PRECISION	a.m.u.	None
partial_charge	DOUBLE PRECISION	e	None
formal_charge	SMALLINT	e	None
charge_group_id	INTEGER	N/A	None
rfos_contribution	DOUBLE PRECISION	kcal/mol	None
solvation_group_id	INTEGER	N/A	None
bound_partners	INTEGER[5]	N/A	None
zmatrix_references	INTEGER[4]	N/A	None
zmatrix_values	DOUBLE PRECISION[3]	Å and °	None

Atom-based metadata are more volatile and less obvious than the simulation metadata in Table 2, particularly for parameters such as “partial_charge”, “formal_charge”, etc. Populating this tier is much simpler in the reference implementation in CAMPARI than using the standalone C++ library because the automated perception and knowledge of, e.g., Z-matrix representations are missing in the latter. A similar caveat applies to naming and ordering conventions, which is why all names are automatically homogenized in CAMPARI, which largely follows the PDB standard, and a consistent ordering is produced for standard entities. This is very helpful when the same system is simulated in two different force fields, for example.

Most of the fields mentioned above are self-explanatory. For the remainder, the details are as follows. The field “sys_seq” differs from “atom_seq” only if the SQL-deposited system is a subset, and in this case, it will refer to the original (complete) system instead. The “rfos_contribution” is a proxy for hydrophilicity and, in CAMPARI, is automatically produced if the ABSINTH implicit solvent model^49,50^ is enabled during deposition. It is the atomic weight factor multiplied by the reference free energy of solvation of the corresponding group whose index is stored in “solvation_group_id”. Bond topology is stored in “bound_partners” (all indices of covalently bound atoms, matching “atom_seq”) and “zmatrix_references”. The latter recapitulate a standard Z-matrix structure and are in this order: indices of reference atoms (matching “atom_seq” if the corresponding atom is present, 0 otherwise) for defining a bond length, a bond angle, and a dihedral angle, whether proper or improper, or a second bond angle, along with the chirality indicator, which is −1, 0, and 1 (0 representing the case of a dihedral angle being used, which captures chirality implicitly). The column “zmatrix_values” gives values for a bond length, a bond angle, and either a second bond angle or a dihedral angle that are representative of the system in question. Lastly, “biotype” is a fixed reference to the role of the atom in known entities, while “molecule_id” distinguishes molecules (molecules connected by cross-links, including disulfide bridges, are treated as separate molecules).

#### Coordinate Tier

The coordinate tier is a system of SQL tables that are named systematically and reference the “sim_id” and “sim_rank” fields of the simulations tier. Each of these tables, which we refer to as snapshot tables due to their name being “snapshots_(sim_id)_(sim_rank)”, contains a reference to the numerical “ens_id” for quicker cross-referencing; see Table 4. This design is chosen because it prioritizes coordinate retrieval speed. We concede that large numbers of tables are unusual in SQL databases. However, because there is never a need to search the space of tables, we do not anticipate that this has adverse consequences on performance, as long as tables can be represented compactly in physical memory. Individual tables tend to map to individual files in PostgreSQL, normally ensuring this compactness. As a result, a practical concern can be that very large (≥1*M*) numbers of tables trigger standard file system performance issues related to the number of files. There is some control on the storage layout, e.g., through “tablespaces,” but this is not explored here.

**Table 4:** Coordinate Tier

Column name	PostgreSQL variable type	Array length	Key status
ens_id	BIGINT	N/A	None
snap_id	BIGINT	N/A	Primary
sim_step	BIGINT	N/A	None
previous_id	BIGINT	N/A	None
previous_rank	INTEGER	N/A	None
previous_snap	BIGINT	N/A	None
sampler_id	SMALLINT	N/A	None
trajectories	(INTEGER, 3× DOUBLE PRECISION)	Dynamic	None
box	DOUBLE PRECISION	12	None

The actual structural data are stored as PostgreSQL arrays of 4-tuples composed of an atom index and the Cartesian *x*-, *y*-, and *z*-coordinates. Both the three box vectors and the formal origin of the simulation system are stored in a 12-vector where the order of Cartesian coordinates is again *xyz* (box vector 1, then box vectors 2, 3, and finally the origin). This is done to accommodate general triclinic containers (without storing the origin, assumptions about alignment have to be made) as well as curved containers (spheres and cylinders). Information about the shape and periodicity is stored in the simulation tier and must be cross-referenced to interpret the box values correctly (CAMPARI uses specific conventions for defining spheres and cylinders in vectors).

Each row in a snapshot table is comparable to the information contained in a single snapshot of an ordinary binary trajectory file. Because the index (normally proportional to simulation step number, “sim_step”) of a snapshot, “snap_id”, is the primary key, accessing an arbitrary snapshot requires only near-constant time . The values in “previous_id” and “previous_rank” allow the construction of the name of the table where the snapshot can be found, which is the geometric parent of the current one. The index of the parent snapshot is stored in “previous_snap”. Taken together, these three fields provide a backward connectivity map that stores where, in a trajectory ensemble, exchanges or replacements took place, which is fundamental for understanding the data contained in such nontrivial trajectory ensembles. Prominent examples are found in replica exchange (swap history) or adaptive sampling (reseeding history). The map is written backward rather than forward because the structure is generally a one-to-many or one-to-one mapping, but never a many-to-one mapping, when looking in the forward direction. Thus, there is at most a single backward mapping per snapshot, which is conveniently stored.

The coordinates are packaged into vectors of a composite data type that contains a running index for atoms (starting at 1, aligned with the “atom_seq” field of the atoms tier) and the *x*-, *y*-, and *z*-coordinates in full double precision. The choice of precision is deliberate, even though in many cases the actual precision of the coordinates will be lower, or such a high precision will not be needed for downstream operations. In order to reduce data transfer times, it might be useful to dynamically reduce the precision already at the SQL level when querying the database.

### Query Implementation

**Figure 2:**
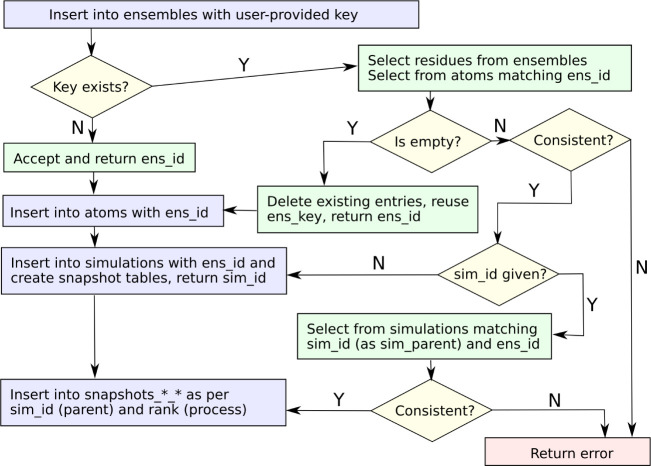
Suggested flowchart for data entry. The first consistency check should verify that the preexisting and deposited systems are identical. If, on the other hand, a system is empty (has no deposited snapshots), it should be overwritten. The second consistency check primarily preserves the integrity of trajectory ensembles when appending existing snapshot tables in parallel. The purple boxes are the steps that can add rows to PostgreSQL tables, i.e., write data.

In the interfaces we have created, we use programmatically generated queries to interact with the PostgreSQL database through libpqxx. The queries follow a logic designed to keep the rate-limiting steps, which we assume will be coordinate deposition and retrieval, as simple as possible. It is important to note that the choice of query logic is not part of the standard. It is precisely one of the strengths of the reliance on SQL that the data can be interacted with in a way that is dependent on the application context. A possible flowchart for data entry might look like the one in Figure 2. The goal of the scheme is to protect the integrity of the database while cross-linking information as much as possible. For example, if exactly the same protein construct is simulated by different groups using different force fields, then they should share a common key. If, however, two different mutants of the same protein are simulated by the same group using the same force field, this is not possible. In the latter scenario, it will be beneficial not only to make the system findable (choice of “ens_key” and data in “sim_summary”) but also to explicitly store related keys in the metadata.

**Figure 3:**
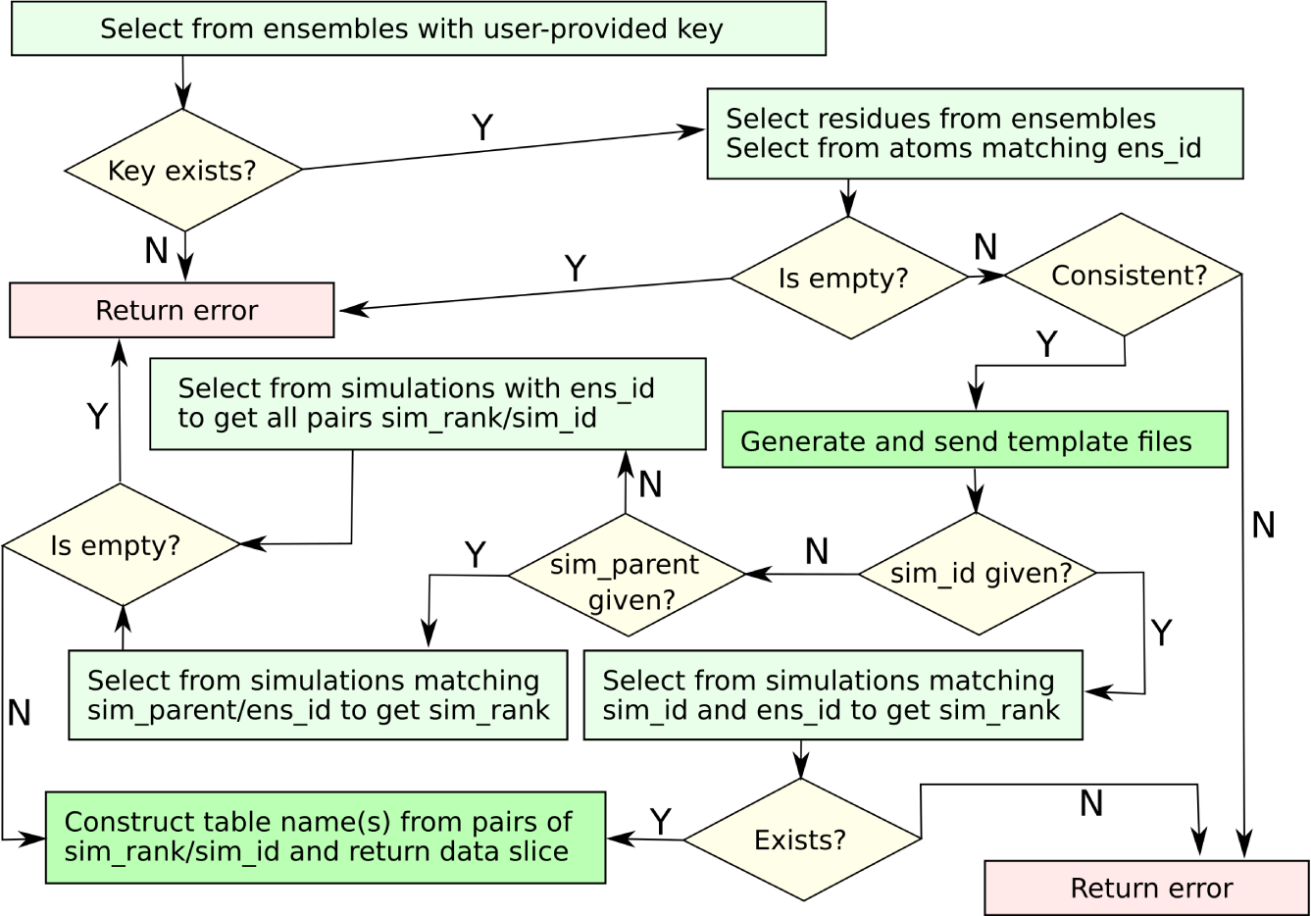
Suggested flowchart for data retrieval. The dark green boxes denote actual results sent to the client. The generation of a structural template requires information from the atoms tier and at least one snapshot table (not shown) to get example coordinates. A Z-matrix template can be generated from the atoms table alone, while the sequence template is contained directly in the ensembles tier. In the routes where more than one simulation is addressed, it is important to sort the tables in a well-defined manner, normally by “sim_parent” and then “sim_rank”. If not, the client cannot request mappable data slices. The final retrieval step omits the interpretation of snapshot index(es) and atom subsets.

When a client is reading from the database, we anticipate that the foremost access reference will be “ens_key”. For example, in our visual interface (see below), the user is greeted with a data entry field and a button to obtain a list of all available keys. With a specific choice of “ens_key”, we can follow a flowchart such as that in Figure 3.

In the following, we provide three example queries. First, the following creates the names of all expected snapshot tables that are associated with a given “ens_key”:

SELECT 'snapshots_' || sim_id || '_' || sim_rank FROM simulations WHERE

ens_id = (SELECT ens_id FROM ensembles WHERE ens_key = 'Beta3S_CHARMM19');

The following statement will instead find a list of suitable simulation and ensemble identifiers based on a text search in the “sim_summary” field:

SELECT ens_id, sim_id, sim_rank FROM simulations WHERE sim_summary

LIKE '%bromodomain%';

The final query retrieves the coordinates of all *C*_α_ atoms for a specific snapshot from a given table in the coordinate tier as rows of *x*, *y*, and *z* values:

WITH hlp AS (SELECT UNNEST(trajectories) AS ixyz FROM snapshots_1_0

WHERE snap_id = 4) SELECT (ixyz).x,(ixyz).y,(ixyz).z FROM hlp WHERE

(ixyz).atom_seq IN (SELECT atom_seq FROM atoms WHERE atom_name LIKE '%CA%'

AND ens_id = 1);

This assumes that a previous query has matched a supplied key with both a numeric “ens_id” (here, 1) and a matching snapshot table (here “snapshots_1_0”). The “UNNEST” function is needed to convert a PostgreSQL vector to rows, while the custom datatype can be indexed as shown (fields “x”, “y”, “z”, and “atom_seq”). Naturally, not all requests are written elegantly as single queries, and in many cases it will be beneficial to rely on functions to structure the queries better and to avoid creating unnecessarily large, intermediate results. The actual execution plan is, in any case, a matter of implementation, and the fine-tuning or even the construction of queries will, in general, not be exposed to the domain scientists accessing the database. This is exactly the role of the implementation, here in both CAMPARIv5, the extracted C++/Python API, and the visual user interface discussed below.

### Performance Evaluation

In this section, we provide brief evidence that there are no inherent scalability issues in writing data to or retrieving data from PostgreSQL databases in the standard defined above. It is important to point out upfront that our focus is not on evaluating the scalability of PostgreSQL itself, except to investigate whether having a large number of coordinate tables can be problematic. Furthermore, we neither claim nor aim to demonstrate that a trajectory database in SQL format outperforms traditional binary trajectory files for typical sequential writing and reading of data.^25^ Superior performance is not expected and does not constitute the objective of our design. There are two main reasons for slowdowns: first, the reference implementation chosen here commits to the database after each incremental write, which is not necessary. In practice, this means that every table row is fully validated and is made visible after each deposition operation. Almost all of the standard options for writing to the database imply a level of safety not seen in binary file formats, mostly to protect database integrity. For example, we could have used a standard libpqxx “work” class, but that would imply that changes are tracked and can be rolled back until a commit, leading to obvious overhead and no speed-up. Second, the coordinates are converted intermittently to a string representation, which is theoretically avoidable but simpler to use because of the composite type in the coordinate tier. This conversion relies on C++’s “to_string” function, which itself is an unnecessarily slow solution.

**Figure 4:**
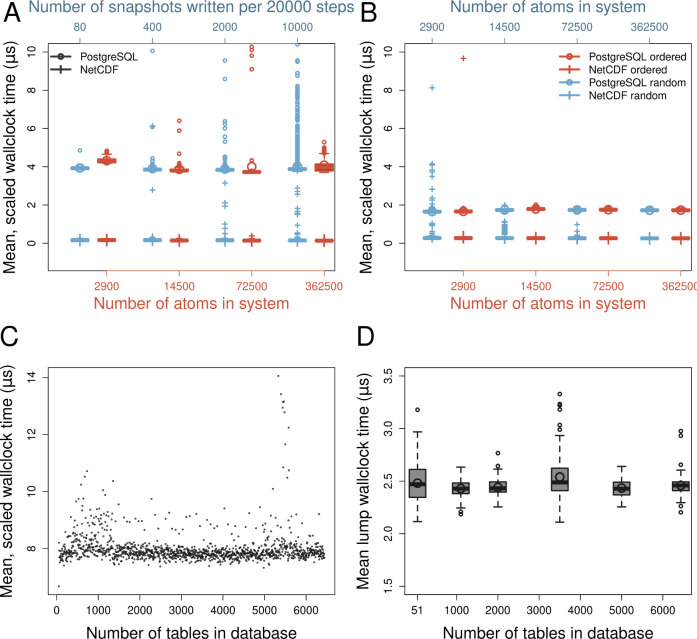
PSQL I/O has the expected constant cost. (A) The times (red, *n* = 199) are measured wall-clock times for entering and exiting the structure write routines in CAMPARI. They are presented as Tukey-rule boxplots with the means given by the large circles. The data as a function of write interval (blue, top axis, *n* = 79, 399, 1999, 9999 from left to right) are included to diagnose potential issues with buffer backlogs, and all used the 14500-atom system. (B) The same as (A) but for reading data either in sequential order (red, *n* = 200) or in random order (blue, *n* = 500). Note that the *x*-axes in panels A and B are logarithmic. (C) The time cost of writing per snapshot and atom (same as A) but for 5 processes accessing the database simultaneously. Every data point is an average of 150 individual snapshot writes. (D) The lump cost of the whole execution: initialization, reading from PostgreSQL, processing, and writing to NetCDF. The data are normalized per snapshot and atom and averaged across 5 processes. Tukey-rule boxplots (*n* = 201) are presented, with the means given by the large circles. Each run used a different set of 5 tables to read from, except for the case of 51 tables (marked on *x*-axis) where always the same set of 5 was used. All data were measured on a desktop equipped with a (6-core) Intel i7-8700 CPU and 16GB of main memory running CAMPARIv5, PostgreSQL 12.12, libpqxx 6.4.7, and NetCDF 4.6.2. The machine used for testing was not fully isolated, meaning that many visible outliers, particularly in (C, D), will be random.

Figure 4A,B demonstrates strong scaling by the normalized time cost per snapshot and atom, of writing (A) and reading (B) being roughly constant across different orders of magnitude of the control parameters. Figure 4C,D emphasizes that, at least in a regime of thousands of tables, our design choice for the coordinate tier has no effect on performance. There are three minor issues to discuss. First, in (A), the write stream can become saturated when a lot of snapshots are written, leading to outliers in both PostgreSQL and NetCDF. Second, there is a constant cost of accessing the database that becomes visible for small systems. This (PostgreSQL, 2900 atoms) is the only systematic deviation from constancy that is not caused by outliers. Third, in (B), random access of snapshots leads to some outliers with NetCDF but not PostgreSQL.

In general, the cost of reading is much more similar to the cost of writing between the two approaches. This is because there is no cost from “committing” the database after each read access. It is still slower than NetCDF because, aside from library-intrinsic reasons, the interface in use retrieves the “coords” entry for a given row as a string that is decomposed and converted using string parsing, whereas NetCDF reads native data types. As mentioned above, this is theoretically avoidable.

### A Visual Interface

Molecular simulation data are, in conjunction with quantitative analyses, often processed visually. This serves the following purposes: to verify that a simulation proceeds as expected, to try to understand correlated motions, or to illustrate and explain analysis results. It is widely accepted that interfaces to structural data in molecular science embed visualizations, for example, in the PDB^48,51^ or the Cambridge Structural Database.^52^ Evidently, the community perceives an intrinsic value in graphically interpreting structural data, and the same idea holds for the mixed structural-dynamic data contained in molecular simulation trajectories. At the public URL https://acgui.bioc.uzh.ch/acgui/, we have set up an example solution for how the standard presented in this manuscript can be utilized in conjunction with a standard web server architecture to serve molecular trajectory data to clients globally (button “Direct SQL”, top bar). We have preloaded the server with a few data sets from both our work and that of D. E. Shaw,^53^ for example, “BETA3S_C19_MD” is the “ens_id” for our well-established simulation data set on a β-sheet miniprotein.^54^ This solution is not part of the standard, and its implementation is out of the scope of the current article (to be presented elsewhere). Based on a user request, it retrieves, interprets, and visualizes molecular simulation data using WebGL (in a heavily modified version of 3Dmol^20^), offering established representations (space-filling, cartoon, etc.) while operating by default in streaming mode (continuously obtaining and deleting data) to protect the memory of the client-side browser. Briefly, the interface offers the following components relying on the PostgreSQL database: a retrieval mechanism for keys based on a text search in “sim_summary”; access to the meta-information for the selected simulations; the ability to subset snapshots arbitrarily; the ability to subset atoms by component (e.g., macromolecules only); the option to download specific selections in all common file formats; alignment of structures; and retrieval of nearby water molecules using arbitrary selections for the reference sets. The last point is often overlooked: the correct alignment of systems containing multiple macromolecules or the identification of proximity based on reference sets requires understanding distance relations in, usually, periodic containers, which is far from trivial, in particular for triclinic boxes.

The system is built with a standard web server architecture inherited from Pharmit^55^ and requires nothing more than a modern browser on the client side. The backend uses custom-built C++ code for interactive operations (queries, metadata collection, template/trajectory retrieval, etc.) and CAMPARIv5 for aspects such as trajectory conversion and analysis. The web server architecture minimizes the software requirements on the client side, which is an explicit design goal.

## Concluding Discussion

We describe here a possible standard to improve the availability of molecular simulation data, in line with FAIR principles. Our goal was to provide not only a standard but also a reference implementation, which is hosted in the established and freely available software package CAMPARI. To this end, we defined a database format, table layout, and query structure for PostgreSQL and an interface from CAMPARI to PostgreSQL relying on the libpqxx library. The choice of tools was motivated by their open-source status and capabilities, which, in the case of PostgreSQL, includes the availability of useful native features (vectors). The performance was shown to satisfy the design requirements, which were to offer access to arbitrary slices of trajectory data with a time complexity that, in practice, is limited by constant factors and the size of the data to be transferred but not by having to scan or search. The reliance on SQL entails, as a primary downside, a fundamental performance hit relative to reading binary files without jumping. Importantly, there is no need for production engines to write directly to SQL: this will normally happen at a later stage and can proceed independently using, e.g., the reference implementation in CAMPARI to perform the conversion. Some obvious benefits offered by SQL are as follows. We can associate and strongly link metadata; we can allow users to design their own search terms to filter information; we can host data from different projects in a consistent environment with automatic backup schemes in place; last, we can rely on the server scalability mechanisms that PostgreSQL offers and that have been demonstrated in practice for extremely large databases like Instagram’s user database.^56^ We provide near-complete interactability with a trajectory database defined in the standard presented here (excluding maintenance and cleaning operations) at the command-line level. To complement this graphically, we also reference a browser-based solution that relies on a server architecture, which will be presented in full elsewhere.

As a standard, our solution can be deployed in different contexts: as a personal data management system, within a locally visible sharing and management platform, such as within companies, academic groups, or university departments, or within a world-visible data sharing initiative. At the single-researcher level, it offers a long-term storage option for data converted from existing binary file formats. Here, it is sufficient that PostgreSQL, libpqxx, and one of the reference implementations run on a local machine. For the other two levels, it will be beneficial to have an embedded, browser-based solution available, and we have presented such an interface here. It must be emphasized that there is no substantial difference in how the standard is used across levels, i.e., the query and retrieval interfaces are modular components not prescribed by the standard. For example, as an addition, a standard web-based SQL interface might be preferred to carry out metadata queries more flexibly. It is, however, important to emphasize that curation is both difficult and important, and this is why we think it is preferable to offer our primary reference implementation in software that offers perception and limited validation in addition to format conversion from and to all common binary file formats.

The few globally visible and active initiatives for sharing simulation data via the web range in scope from downloadable archives of files, e.g., Misato,^57^ to sophisticated web servers with strong metadata association like GPCRmd^28^ or BioExcel COVID-19.^30^ Different web servers have implemented their own solutions, which are often infrastructure-dependent, but to the best of our knowledge, they all serve individual files. In the example of GPCRmd, the authors have created a very complex layer of metadata that is somewhat topic-specific. They have converted coordinates to padded binary files (for constant-time access in the snapshot dimension), and they have implemented everything in PostgreSQL accessed by a web server architecture (see SI in ref (28)). It differs conceptually from our approach here in two major ways: first, much of the metadata is not directly related to simulations but to context, such as papers, users, and sequence annotations; similar to what is found in PDB/mmCIF metadata. All of this type of information would go into the “sim_summary” field of the simulations tier. Second, we retain no original files as part of the standard, whereas GPCRmd stores filenames and paths, thus making some queries, such as those for detailed simulation settings, impossible without leaving the SQL environment. It is noteworthy that, for serving heterogeneous collections of files, NoSQL solutions have been advocated as superior.^58^ The differences clearly result from different aims: GPCRmd tries to make comparative analyses of evolutionarily related families of proteins possible while strongly linking simulation data with the experimental backdrop. Our goal, in contrast, is to overcome the weaknesses of existing file formats while improving the FAIR compliance of the stored data.

It is by now well appreciated in the field that FAIR principles are worthy guideposts for managing and sharing simulation data.^1^ Globally visible servers, especially those with a topical focus or high level of standardization,^28−30,58^ have been set up because sharing repositories in isolated binary formats is insufficient: it creates too large a gap between data and metadata. The level by which a server circumvents the need for specialized tools on the user side differs because, and this is universally true, a server cannot operate safely and at scale while also anticipating every possible task anonymous users might want to carry out. Thus, as a starting point, the data should be hosted in a way that makes them directly viewable and analyzable in the ways that the practitioners in the field are used to, i.e., using molecular viewers and performing field-specific analyses (such as RMSD after 3D alignment). This function is performed by most of the existing servers, to different extents, in particular regarding analysis, including by the graphical user interface we developed. It is not a necessary element of a data management solution on a smaller scale, however, which is why we separate the trajectory storage standard from the question of how to serve these data. Importantly, during the development, we realized that the more knowledgeable the software is regarding the systems being simulated, the more useful the interface will be. Aspects such as the ability to compute periodicity-corrected distances in triclinic boxes or knowledge of the native bond topologies of biopolymers are built into software packages such as CAMPARI but absent from tools that treat coordinate data as simple data matrices.

While public servers provide implementation, data, and analysis jointly, we argue that these aspects benefit from remaining modular, as the long-term hosting of community data must generally be addressed by larger-scale consortia. Importantly, the very recent past has seen increased recognition of the need to improve the findability and interoperability aspects of molecular simulations by strengthening metadata links and recognizing the fractured nature in which they are stored.^3,59−61^ The availability of several competing tools, with partially unique and partially overlapping functionalities, will be an asset in allowing data hosting organizations to choose the solutions most apt for the hosting architecture they employ.

To us, one fundamental challenge in the field of stochastic simulations of biomolecules is to delineate raw observations, which are extremely high-dimensional, from the analyses performed on them that end up in published papers. Unlike in other fields, it is never the case that a molecular simulation, recognizing it as the “experiment’s” raw data, is analyzed comprehensively, which is an inherent property of their nature. The very long MD trajectories of D. E. Shaw generated around 2010^53,62^ using custom hardware^63,64^ are a case in point. They have provided a community-wide benchmark set by finding reuse as either raw or reference data in numerous publications by unrelated authors.^65−70^ Undoubtedly, the high prominence of the reference publications, their boundary-pushing nature while still being conventional MD, and the straightforward availability of the raw trajectories all contributed favorably to this. Yet, from our own experience of working with them,^71−73^ these data would have benefited from tightening the link between metadata and data, for example, to highlight unusual conditions (temperature), mutations, etc. In contrast, the level of reuse of nonstandard simulations, such as nontrivial, multireplica runs, force-biased methods, etc., documented in the literature is negligible, at least across research groups. As a first step, the standard introduced here does allow traversing some of these trajectory ensembles cognizant of their nature. Of course, the fitness of existing data to answer specific hypotheses is unknown. Thus, these often hidden data^3^ might ultimately be more useful for scientists interested in understanding the models they use at scale, rather than answering system-specific questions. One potential use of the standard we present here is to join analyses from proteins in the same family by transcribing them to a unified, low-level representation such as backbone atoms of all homologous positions. This can all be done within the PostgreSQL database with the interface we provide.

## Data Availability

The primary implementation described herein is part of CAMPARIv5, which is available freely and as open-source software from https://sourceforge.net/projects/campari/ and was used to generate the performance benchmarks in Figure 4. The C++ functions for managing the database have been exported into a standalone version, fully documented and with Python bindings; see https://gitlab.com/CaflischLab/TrajPSQLmod. The authoritative version of the standard will be maintained as part of CAMPARI’s documentation; see http://campari.sourceforge.net/V5.
